# Simplifying Data Analysis in Biomedical Research: An Automated, User-Friendly Tool

**DOI:** 10.3390/mps7030036

**Published:** 2024-04-24

**Authors:** Rúben Araújo, Luís Ramalhete, Ana Viegas, Cristiana P. Von Rekowski, Tiago A. H. Fonseca, Cecília R. C. Calado, Luís Bento

**Affiliations:** 1 NMS—NOVA Medical School, FCM—Faculdade de Ciências Médicas, Universidade NOVA de Lisboa, Campo Mártires da Pátria 130, 1169-056 Lisbon, Portugal; 2 CHRC—Comprehensive Health Research Centre, Universidade NOVA de Lisboa, 1150-082 Lisbon, Portugal; 3 ISEL—Instituto Superior de Engenharia de Lisboa, Instituto Politécnico de Lisboa, Rua Conselheiro Emídio Navarro 1, 1959-007 Lisbon, Portugal; 4 Blood and Transplantation Center of Lisbon, IPST—Instituto Português do Sangue e da Transplantação, Alameda das Linhas de Torres 117, 1769-001 Lisbon, Portugal; 5 iNOVA4Health—Advancing Precision Medicine, RG11: Reno-Vascular Diseases Group, NMS—NOVA Medical School, FCM—Faculdade de Ciências Médicas, Universidade NOVA de Lisboa, 1169-056 Lisbon, Portugal; 6 ESTeSL—Escola Superior de Tecnologia da Saúde de Lisboa, Instituto Politécnico de Lisboa, Avenida D. João II, Lote 4.69.01, 1990-096 Lisbon, Portugal; 7 Neurosciences Area, Clinical Neurophysiology Unit, ULSSJ—Unidade Local de Saúde São José, Rua José António Serrano, 1150-199 Lisbon, Portugal; 8 Institute for Bioengineering and Biosciences (iBB), The Associate Laboratory Institute for Health and Bioeconomy–i4HB, Instituto Superior Técnico (IST), Universidade de Lisboa (UL), Av. Rovisco Pais, 1049-001 Lisboa, Portugal; 9 Intensive Care Department, ULSSJ—Unidade Local de Saúde São José, Rua José António Serrano, 1150-199 Lisbon, Portugal; luis.bento@ulssjose.min-saude.pt; 10 Integrated Pathophysiological Mechanisms, CHRC—Comprehensive Health Research Centre, NMS—NOVA Medical School, FCM—Faculdade de Ciências Médicas, Universidade NOVA de Lisboa, Campo Mártires da Pátria 130, 1169-056 Lisbon, Portugal

**Keywords:** biomedical research, machine learning, LLM models, high dimensional data analysis

## Abstract

Robust data normalization and analysis are pivotal in biomedical research to ensure that observed differences in populations are directly attributable to the target variable, rather than disparities between control and study groups. ArsHive addresses this challenge using advanced algorithms to normalize populations (e.g., control and study groups) and perform statistical evaluations between demographic, clinical, and other variables within biomedical datasets, resulting in more balanced and unbiased analyses. The tool’s functionality extends to comprehensive data reporting, which elucidates the effects of data processing, while maintaining dataset integrity. Additionally, ArsHive is complemented by A.D.A. (Autonomous Digital Assistant), which employs OpenAI’s GPT-4 model to assist researchers with inquiries, enhancing the decision-making process. In this proof-of-concept study, we tested ArsHive on three different datasets derived from proprietary data, demonstrating its effectiveness in managing complex clinical and therapeutic information and highlighting its versatility for diverse research fields.

## 1. Introduction

Biomedical research is fundamental to advancing medical knowledge, driving innovations in healthcare, and developing new therapeutic interventions. It enables the scientific community to understand disease mechanisms better, identify potential targets for treatment, and evaluate the efficacy and safety of novel drugs and procedures, thereby directly contributing to improving patient outcomes and public health [1,2]. The credibility and reliability of results obtained hinge on the ability to draw statistically supported conclusions about the target investigation, e.g., including the search for diagnostic biomarkers, or the evaluation of the efficacy of medical treatments [3]. One critical factor influencing the validity of these conclusions is the characteristics of the populations being studied [4]. 

Baseline characteristics, such as age, gender, comorbidities, and disease types and severity, play a pivotal role in determining the efficacy of biomarkers’ discovery and treatment outcomes, among others. Imbalances in these characteristics across the study and the control groups may introduce confounding variables, potentially shuffling the interpretation of the results obtained [5]. One way to address this, especially when dealing with the comparison of diverse groups of individuals is population normalization, is to ensure that any observed differences between study groups can be confidently attributed to the intervention rather than pre-existing disparities in participant characteristics [6]. To achieve this, the compilation and curation of datasets stands as a cornerstone of the investigative process. 

Another critical point is the management of missing values, as incomplete data can bias the analysis, affect the statistical power of the study, and ultimately lead to misleading conclusions. Properly addressing missing data ensures the robustness and validity of research findings, enabling accurate interpretation and generalization of results. Techniques to manage missing values range from simple imputation to more complex statistical models, each carrying different implications for the integrity of the dataset and the reliability of subsequent findings [6,7,8].

Therefore, the journey from raw data and/or biological samples to refined datasets encompasses a methodical progression through which researchers carefully glean and refine data. This time-consuming process ensures the integrity and quality of data, which in turn underpins the reliability of research outcomes. This foundation of rigorously preprocessed and robust datasets not only fortifies the research outcomes but also sets the stage for groundbreaking advancements in the identification and utilization of biomarkers across various biomedical fields [9].

The main examples of applications that we have chosen to focus on relate to the discovery of biomarkers based on omics sciences. This choice is motivated by the potential of these biomarkers to revolutionize diagnostics and therapeutics [10]. Omics sciences, including genomics [11], transcriptomics [12], proteomics [13], and metabolomics [14], offer an holistic and comprehensive analysis of biological systems, unraveling the complexities of life at the molecular level. This approach provides insights into the dynamic interactions within cells and organisms, facilitating a deeper understanding of disease mechanisms, physiological responses, and environmental effects on biological functions. 

The comprehensive data generated by omics technologies have been instrumental in the discovery of biomarkers for disease diagnosis, prognosis, and therapeutic targeting, including, e.g., in studies of cancer [15], genotoxicity [16], organ rejection [17,18], and infections [13,19]. By integrating data across different omics layers, researchers can construct a more complete picture of biological processes, thereby enhancing our understanding of health and disease [20,21]. 

However, the field of omics also faces challenges, like high costs, complexity, and variability in human studies. To counteract this, there has been a focus on omics sciences, bolstered by advancements in machine learning [22,23] and large language models (LLMs) [24,25,26]. These technologies have demonstrated significant potential for processing and interpreting the extensive datasets produced by omics techniques [27]. This development underscores the imperative to provide to these techniques the highest quality and most meticulously curated databases possible.

In response to the critical gap in the availability of user-friendly, efficient, and broadly accessible data analysis resources in biomedical research, we developed this proof-of-concept work and its accompanying tool. Recognizing the necessity for a solution that demands minimal input from the user and no prior knowledge of data analysis or statistical methods, this tool was designed to be straightforward and instructive, even on older computer systems with limited capabilities. Unlike many commercial offerings that are often complex and resource-intensive, our tool stands out for its simplicity, speed, and the absence of unnecessary features.

Building on this, the motivation behind the creation of this new tool was the lack of open-source tools capable of seamlessly integrating advanced analytics and educational features—coupled with the latest artificial intelligence (AI) technologies, such as OpenAI’s GPT-4, for refined data interpretation. This tool was, therefore, developed to fill a significant gap by providing a broadly accessible data analysis resource in biomedical research. It is intended that this tool enables the normalization of complex datasets with minimal input from the user and minimal knowledge of data analysis or statistical methods. Our aim is to make the process of data collection, preparation, and analysis more effective and accessible, thereby facilitating a more inclusive and comprehensive approach to biomedical research.

## 2. Methods

### 2.1. The User Experience—An Overview

ArsHive offers a comprehensive yet user-friendly experience for biomedical informatics research. Upon launching the software, users are greeted with a clean interface, where the primary step involves loading a dataset, either in the comma-separated values (CSV) or Excel formats. After uploading a dataset, users can select from six different normalization algorithms that automate the process and generate a comprehensive report on the data. These algorithms offer options for sample equalization, proportion adjustments, and hybrid methods for complex data manipulation. Alternatively, users have the option to calculate statistics either for selected or all variables in the dataset, enabling a quick review of the data and its relevant statistics without undergoing any normalization. 

**Figure 1:**
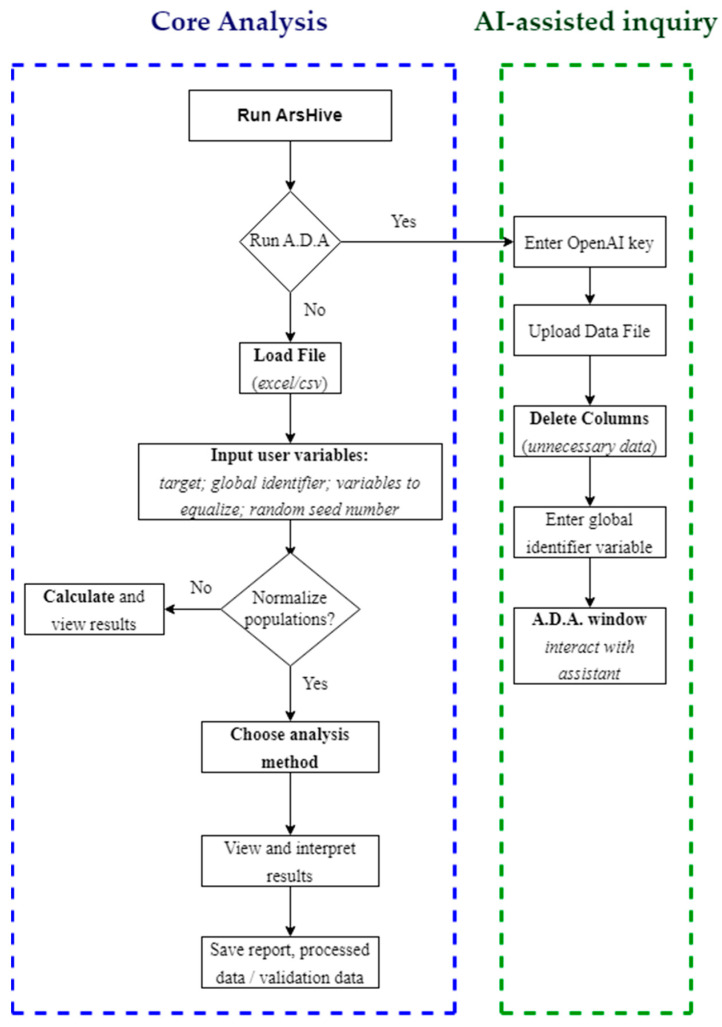
User experience flowchart: dashed blue rectangle outlines the main functions of ArsHive, whereas the dashed green rectangle indicates the AI-assisted path.

Figure 1 presents a streamlined flowchart that traces the user’s journey within ArsHive, from launching the application to obtaining and interpreting results. This visual tool illustrates the critical decision-making junctures—such as whether to engage the A.D.A. tool for enhanced analysis and when to choose the analysis method. The flowchart emphasizes the software’s interactive nature, where users can toggle between different analytical paths, making informed choices, like whether to normalize their dataset or proceed directly to analysis. A.D.A.’s integration within this process is clearly marked, indicating where users can opt for a chat-based interactive session with the AI assistant for additional support. This encapsulates a user-centric approach, providing guidance at each step while allowing flexibility to explore the data in a way that best suits their research needs.

### 2.2. Enhancing Traditional Sampling through ArsHive

Researchers have access to a range of sampling methods, each tailored to different types of studies. Among the probability sampling methods, simple random sampling selects subjects from a larger population entirely at random. Stratified random sampling divides the population into distinct subgroups, or strata, ensuring representation across key variables and providing balanced and unbiased estimates. Systematic sampling selects subjects at regular intervals from an ordered list, offering simplicity and efficiency. Conversely, clustered random sampling involves dividing the population into clusters, such as geographic areas, and then randomly selecting entire clusters for study, which helps reduce costs and logistical challenges [28]. 

For non-probability sampling, convenience sampling involves selecting the most easily accessible subjects, although this may not represent the general population. Targeted sampling relies on the researcher’s judgment to choose participants thought to be most relevant to the study objectives. Snowball sampling is used to access populations that are difficult to reach; it starts with a small group of known individuals (often called seeds), who then recruit further participants [28]. 

Stratified sampling, perhaps the most commonly used method among researchers, has proven particularly beneficial in medical research, as demonstrated in longitudinal studies [29], facilitating a more representative and reliable data collection. Nonetheless, there is an acknowledged need for better understanding and application of data balancing among medical researchers [30]. Given this gap, the authors felt compelled to create ArsHive, a tool that offers robust population normalization, thereby enhancing the reliability of research outcomes and providing educational benefits to its users. While traditional sampling methods offer robust frameworks for research design, they can be limited when applied to pre-existing datasets that were not initially collected with these methods, or when the data inherently contains biases that affect study outcomes. 

In response, ArsHive was developed to extend these traditional methods into the realm of post-collection data normalization. For instance, our ‘equalize samples’ method functions as an automated version of simple random sampling, where it randomly selects samples with the goal of equalizing the number of samples across each subpopulation of the target variable. Furthermore, ArsHive’s more sophisticated algorithms could be seen as turbocharged versions of stratified sampling. They dynamically adjust the sizes and proportions within subpopulations, ensuring that the dataset achieves a balance similar to what would be expected if stratified sampling had been applied at the data collection stage. This capability is particularly vital in biomedical datasets, where the representativeness and balance across key variables can significantly impact the validity of research outcomes. By incorporating these advanced features, ArsHive provides researchers with powerful tools to refine their data post hoc, maintaining the integrity and enhancing the reliability of their research findings.

### 2.3. Software Conceptualization and Validation

The development and validation of ArsHive involved a strategic approach to dataset selection, employing both proprietary and open access resources to ensure robustness and comprehensive functionality. Early testing was conducted using large publicly available datasets, as detailed in Table 1, to verify the tool’s readiness and effectiveness, in which the automated statistical tests implemented, summarized in Table 2, played a crucial role in the data analysis and normalization. Regarding the conceptual foundation of this tool, it was established using three proprietary biomedical datasets. These datasets, detailed in Table 3, were carefully anonymized, randomized, and algorithmically modified to simulate real patient data without compromising privacy. Their selection was based on varying sizes and complexities to address specific development and testing needs, a rationale that will be elaborated upon in Section 2.7 and Section 3.3, particularly in relation to the software’s optional companion application.

During the initial development phase, one of the significant challenges addressed was the normalization of data. Biomedical datasets often contain inherent imbalances and missing data points due to selective data acquisition strategies, where not all tests are administered to all patients to manage costs and avoid unnecessary procedures. Such issues necessitate sophisticated normalization strategies, which ArsHive addresses using advanced algorithms for imputation and normalization, including techniques, like mode, mean, forward and backward filling—methods that manage gaps by using subsequent or previous data points, respectively. This not only ensures comprehensive data integrity but also helps maintain the utility of the dataset by minimizing biases that could influence research outcomes.

Following this, ArsHive underwent rigorous testing with large datasets from repositories with open access, as outlined in Table 1. This testing phase included a wide range of patient data, such as demographic details, clinical and vital signs, treatment regimes, genomic information, and metabolites, among others. The aim was not only to ensure the tool’s readiness for real-world application but also to validate its effectiveness and functionality across diverse biomedical datasets—encompassing both numerical and categorical data types, while acknowledging that imaging and continuous signals were beyond the scope of this initial validation.

The selection of open access datasets for this crucial phase was driven by their availability and the opportunity they presented for a comprehensive validation of ArsHive’s performance across varied data complexities. Specific benchmarks for this phase included monitoring error rates (e.g., errors found and reported per iteration), and identifying any unexpected bugs, measuring processing times, and verifying the accuracy of data normalization and analysis outputs, with early results showing promise, confirming the tool’s adaptability and potential for wide-ranging applications in biomedical research. Despite encountering a few anticipated bugs, indicative of the inherent diversity and curation levels of the datasets tested, these initial trials were instrumental in identifying areas for improvement. Subsequent enhancements to ArsHive have been targeted at increasing its capacity to accommodate a broader array of data types, further cementing its role as a versatile and invaluable asset for the scientific community.

### 2.4. Behind the Scenes: How Our Algorithms Work

The integrity and quality of data analysis are vital in biomedical research because they directly influence the reliability and applicability of research findings. Ensuring data integrity means that the conclusions are based on accurate and consistent information, crucial for making informed clinical decisions. Similarly, high-quality analysis guarantees that the results are derived from well-structured and comprehensive evaluations, reducing the risk of errors or biases that could compromise the study’s validity. Particularly when analyzing populations to predict or discriminate based on specific target variables, the integrity and quality of data underpin the development of robust algorithms, ensuring that the outcomes can genuinely inform clinical practices and further research.

To address this, the data are adjusted automatically, for any dataset size, between the participants of a study and a control group, using pre-selected variables per the user command, through a series of sophisticated algorithms, effectively normalizing the population. Categorical data are presented as absolute frequencies and percentages, with continuous variables expressed by their medians and interquartile range (25th percentile–75th percentile). Details about the statistical tests and their applied logic can be found in Section 2.6. By automating the various parameters and population variables (clinical, therapeutical, or others), it is intended to deliver a user-friendly experience while maintaining scientific rigor. 

**Figure 2:**
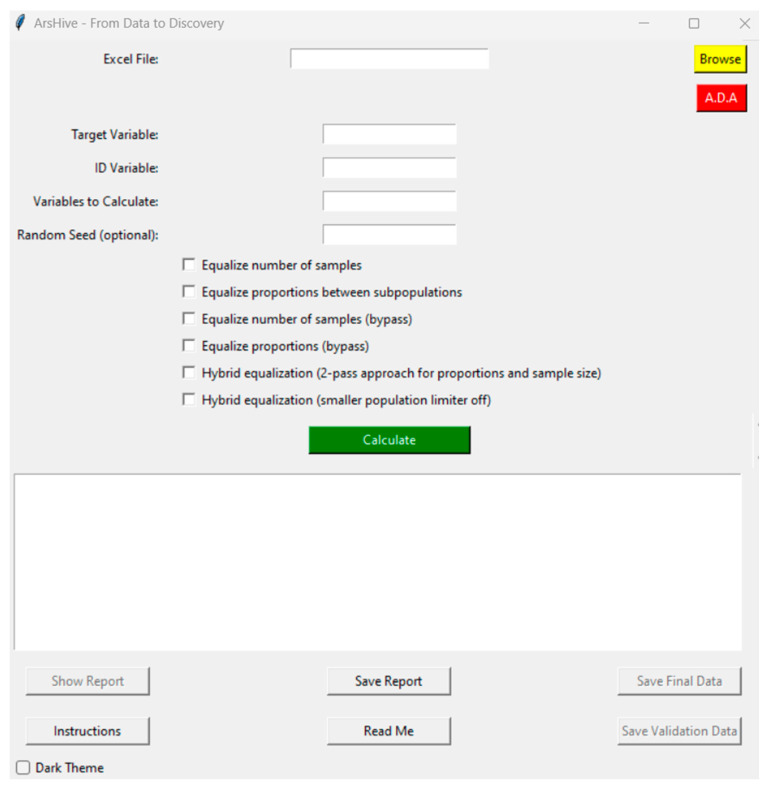
Main GUI of ArsHive, displayed in its default (light) theme.

Figure 2 showcases ArsHive’s main graphical user interface (GUI), designed for ease of use with minimal user input. Key features include a prominent yellow ‘Browse’ button for loading datasets in .csv (comma-separated values) or .xls (Excel) formats. The GUI is structured to prompt users for essential inputs: the target variable for the study (e.g., infection, death, organ failure, etc.), a unique identifier variable for each sample (typically an anonymized number or code in human studies), variables for normalization, and an optional random seed number (starting from 1) to ensure replicability of findings. The interface also includes checkboxes for selecting among the main algorithms offered by ArsHive, facilitating user control over data analysis processes. Notable among other interface elements are a large green button named ‘Calculate’, which activates ArsHive’s suite of statistical tools—usable with or without prior normalization—and a red button named ‘A.D.A.’, linking to the AI companion tool, powered by OpenAI’s GPT-4 turbo. Additional auxiliary buttons provide access to functionalities, such as report generation, data export for training or validation, and comprehensive ‘Instructions’ and ‘Read Me’ guides for detailed usage information.

For clarity, whenever the term ‘equalize’ is used, it refers to normalizing variables across different population groups, including both demographic and clinical/therapeutic variables. It is important to note that ‘equalize’ and ‘normalize’, or any variation of the same, can be used interchangeably in this context.

The ‘equalize samples’ algorithm adjusts the dataset so that each subpopulation of the target variable has the same number of samples. This is particularly useful when there are uneven sample sizes that could introduce bias into the analysis. Its counterpart, ‘equalize proportions’, ensures that the proportions of categorical and continuous variables are consistent across different subpopulations. This method maintains the internal structure of each subpopulation while achieving balance. Both of these functions have a ‘bypass’ mode, which allows for more aggressive normalization of data. In this mode, even the smallest subpopulation (a population can have up to 15 subpopulations, the limit imposed by the authors), can be subjected to equalization, potentially leading to a more uniform distribution of variables but at the risk of reducing the representation of rare categories.

The final and most sophisticated method available in the software is a hybrid normalization function, which we’ll refer to as ‘advanced equalization’. This approach combines the principles of both sample and proportion normalization without strictly limiting adjustments in smaller subpopulations. This is particularly useful when the smallest subpopulation is not significantly smaller than the others, as it allows for further fine-tuning of the dataset without excessively diminishing its size. To illustrate the application of this last function, let’s consider, in Algorithm 1, a pseudocode example that reflects the complexity of its operations while safeguarding the proprietary methodology.
**Algorithm 1.** Algorithm (simplified for visualization) of the advanced equalization function.
Input: Dataset, TargetVariable, VariableList, MinThresholdOutput: EqualizedDataset
Begin    Initialize equalized dataset as empty    Calculate initial subpopulation sizes and proportions for TargetVariable    Set target size to the size of the smallest subpopulation 
   For each variable in VariableList do     Determine the impact score based on deviation from expected proportions    End For 
   Sort VariableList by descending impact scores 
   While subpopulation sizes differ from target size do     For each variable in VariableList do       Adjust subpopulation proportion to match target proportion       If subpopulation size < MinThreshold then        Protect subpopulation from further reduction       End If     End For     Recalculate subpopulation sizes and proportions    End While 
   Output the equalized dataset  End

In the context of the ‘advanced equalization’ algorithm, the ‘Dataset’ refers to the comprehensive set of data points under analysis, each encompassing a multitude of attributes, including the pivotal ‘TargetVariable’. This ‘TargetVariable’ is the principal variable under scrutiny, often being the outcome or characteristic of primary interest in biomedical investigations. Alongside this, the ‘VariableList’ encompasses a number of variables that are considered for their potential influence on the ‘TargetVariable’, adjusted during the normalization to ensure a balanced representation across the various subpopulations within the ‘TargetVariable’. The process is governed by a ‘MinThreshold’, a safeguarding parameter that ensures the preservation of smaller, possibly vulnerable, subpopulations by preventing their reduction beyond a critical limit. 

The culmination of this algorithmic process is the ‘EqualizedDataset’, a refined version of the original dataset, which has undergone systematic adjustments to the sample sizes and proportions of the ‘VariableList’ with the aim of establishing a dataset conducive to unbiased analytical interpretations.

### 2.5. Handling Missing Data

Before the application of statistical tests, ArsHive tackles the issue of missing values with strategies specifically designed for the data type. For categorical variables, missing entries are imputed with the mode, thus preserving the variable’s distribution pattern. This method is applied uniformly to all categorical variables, ensuring consistency regardless of the proportion of missing data. For continuous variables with less than half the data missing, a discerning imputation technique is employed. The tool assesses the variable’s distribution and employs the most statistically appropriate method for imputation, be it the mean, median, or a model-based approach. This careful consideration helps maintain the variable’s authentic properties and reduces potential biases in the analysis.

Nevertheless, it is crucial to acknowledge that any imputation method brings inherent assumptions into the dataset. While substituting missing values with modes or means can lead to a more complete dataset, such alterations might influence the data’s variance and the relationships between variables. As such, researchers are advised to be mindful of the implications of imputed values and to interpret results with due diligence.

### 2.6. Statistical Test Selection 

ArsHive is equipped with a robust statistical analysis module that intelligently discerns the appropriate tests to apply based on the characteristics of the variables at hand. Initially, variables are classified as either categorical or continuous. 

For most categorical variables, a binary classification is assigned, since only two unique categories/groups are exhibited. The analysis of categorical data involves the chi-square (χ^2^) test to assess independence within contingency tables. In instances where the contingency table contains cells with low expected frequencies—which could compromise the validity of chi-square results—Fisher’s exact test is deployed, providing a more accurate evaluation of inferential statistics.

For continuous variables, a normality check is initiated using the Shapiro–Wilk test. If the distribution of the variable does not significantly deviate from normality (*p*-value > 0.05), it is considered normal; otherwise, it is treated as non-normal. Following the normality assessment, the software tailors its approach based on the needed comparisons.

- **For comparisons between two independent groups:**
  - If the continuous variable is normally distributed, an independent samples *t*-test is conducted to compare the means of the two groups.
  - For non-normally distributed data, the Mann–Whitney U (MWU) test is utilized as a non-parametric alternative.
- **For comparisons between three or more independent groups:**
  - ANOVA is the test of choice for normally distributed data, and is used to compare means across the multiple groups. After performing the ANOVA test, post hoc analyses with Tukey’s test can be used to determine pairwise comparisons between all categories of the target variable and to identify which are significantly different from each other.
  - The Kruskal–Wallis one-way ANOVA test is employed as a non-parametric counterpart to ANOVA when the data is non-normal. Post hoc analyses can also be performed in this case.

Upon finalizing the statistical tests, *p*-values are reported, serving as a quantitative measure of significance for the obtained results. The findings are compiled into the report, which offers a comprehensive and enlightening overview of the data analysis, equipping researchers with valuable insights. A summary of the available statistical tests, at the time of writing, can be consulted in Table 2.

### 2.7. Introducing A.D.A.—A Large Language Model Companion Application 

Nestled within our software suite is A.D.A. (Autonomous Digital Assistant), a fledgling yet promising companion application rooted in OpenAI’s large language model. A.D.A. is not just a tool; it is a virtual partner designed to perform a multitude of tasks. Through a user-friendly interface, researchers can directly interact with A.D.A., leveraging its capabilities to parse complex datasets, which are fed to it by the user, and to guide less experienced users through the intricacies of data analysis, suggest methodologies, and provide educational insights into the data it processes. In Algorithm 2, a high-level view of how A.D.A. is processed within our tool can be observed.
**Algorithm 2.** A.D.A interaction process within ArsHive.
**Input:** User-Provided Data File (Excel or CSV format)**Output:** Chat Window with Loaded Dataset
Begin    Initialize ArsHive Software    A.D.A. button is displayed on ArsHive Software Interface 
   When A.D.A. Button is Clicked:       Prompt User for OpenAI API Key       Initialize OpenAI API with Provided Key 
      Open Data Preprocessing Window       Display Options to Upload Data File (Excel or CSV)       User Uploads File       Load Dataset into A.D.A. 
      Calculate Token Count and Associated Cost for Dataset       Display Token Count and Cost in Preprocessing Window 
      Allow User to Modify Dataset if Needed (e.g., Delete Columns)       Confirm Final Dataset for Analysis 
      Transition to Chat Window       Display Summary of Dataset (Variables, Global Identifier, Token Cost)       Initialize Chat Session with GPT-4-turbo Model       Load Dataset Context (Variables, Token Information) 
      User Interacts with A.D.A. through Chat Window       Send Queries Related to Dataset       Receive Responses from A.D.A. based on GPT-4-turbo Model 
      Maintain Session History for Continuous Conversation Context       Offer Option to Save Session Transcript 
   End A.D.A. Interaction on User Command or Window Closure  End

In the technical framework of A.D.A., we have integrated the ‘gpt-4-0125-preview’ model of OpenAI’s GPT-4 series, specifically chosen for its advanced features and capabilities. This iteration of GPT-4, known as ‘gpt-4-turbo’, stands out for its extended context window and updated training data. One of the pivotal reasons for selecting this particular model is its enhanced efficiency and accuracy. The latest GPT-4 model addresses the issue of laziness, where the model may previously not have completed a task comprehensively. This improvement means that A.D.A. is less prone to generating erroneous responses—thereby offering more reliable and accurate assistance to researchers. The faster processing speed of this model variant also contributes to a smoother user experience, crucial in fast-paced research environments. While this advanced model brings numerous benefits, it is important to acknowledge that certain limitations still exist, particularly regarding the direct execution of certain tasks due to OpenAI’s application programming interface (API) constraints. These limitations are explored in Section 3.3, with this section delving into the balance between the model’s capabilities and its constraints, ensuring readers have a comprehensive understanding of A.D.A.’s operational (and optional) framework within our software suite.

### 2.8. Quality Assurance and Open Source Philosophy

In the dynamic landscape of software development, particularly in the realm of biomedical informatics, the role of rigorous quality assurance and a peer-review-based approach cannot be overstated. For our comprehensive software suite, ArsHive, which includes the A.D.A. assistant, these aspects are not mere afterthoughts but integral components of our development principles. In an industry often pressured by tight deadlines and ambitious goals, we place paramount importance on meticulous testing and iterative refinement to ensure the reliability and efficacy of our tools.

From the start, ArsHive has been developed with a focus on diligence and adaptability by a small, dedicated team. Our method involves regular reviews and a willingness to reconsider even established ideas for better outcomes. We are moving towards expanded testing in the wider scientific community, adopting a thorough test-until-it-breaks approach for enhancing our algorithms. Future plans include making the development more collaborative by opening a GitHub repository to invite wider contributions, fostering innovation and software robustness through diverse insights. Our aim is for ArsHive and A.D.A. to either stand alone or integrate with existing open-source tools, enhancing synergy within the biomedical research ecosystem. This approach is designed to improve the accessibility and impact of advanced data analysis techniques. 

**Figure 3:**
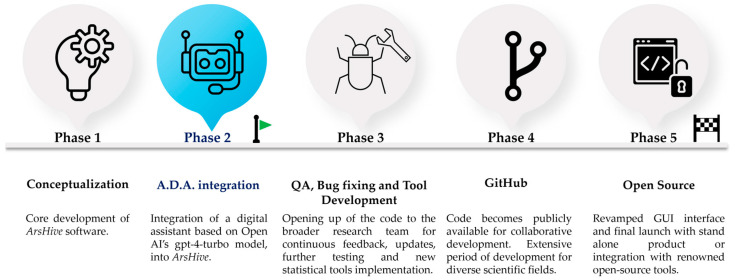
Roadmap for the development of ArsHive software version 0.90.

Figure 3 illustrates the comprehensive roadmap for our tool, delineating its development across five main phases. This progression encompasses the initial conceptualization and current integration of an AI companion tool, marking the tools’ evolution. Subsequent phases—phase 3 through phase 5—focus on implementing significant milestones and new tools, with the latter stages dedicated to broadening accessibility by releasing the tool as open-source code to the wider community.

## 3. Results and Discussion

As highlighted in Section 2.4, ArsHive offers a multifaceted approach to managing biomedical datasets, providing both fundamental and advanced data manipulation techniques. This section offers a condensed review of its capabilities, emphasizing the software’s adaptability for various research requirements.

### 3.1. Different Algorithms for Different Needs

ArsHive’s intuitive design prompts users to define essential variables upon dataset upload. These variables range from mandatory entries, such as the target variable, to optional ones, like a random seed number for consistent reproducibility. Users can then opt for basic statistical analysis or explore the suite of equalization algorithms available to address biases inherent in sample sizes or variable proportions within subpopulations.

For instance, the ‘equalize samples’ algorithm is pivotal when a user needs to address sample size bias across target variable subpopulations without compromising the integrity of smaller, potentially critical subpopulations. As for the complementary bypass function, it allows for a more egalitarian form of equalization, extending the reach of the process to encompass all subpopulations. In the end, the choice of equalization technique is determined by the study’s unique demands and the value of maintaining diversity within the dataset.

ArsHive distinguishes itself within the data analysis landscape with its commitment to open-source distribution and user-centric design. It contrasts with proprietary tools, such as IBM SPSS, XLStat, and GraphPad Prism, by offering the following:

- Open-Source Accessibility: No cost, and the open code enhances transparency and fosters a collaborative improvement environment.
- User-Friendly Interface: Simplified inputs enable ease of use for users with varying expertise levels.
- Automated Data Handling: Pre-configured methods for identifying variable types and imputing missing values (mode for binary data and mean for continuous data) facilitate data preparation.
- Exhaustive Reporting: Detailed reports elucidate findings without overwhelming users.
- Data Integrity Checks: Ensures consistency between datasets before and after equalization processes.

ArsHive’s algorithmic precision is the cornerstone of its design, significantly reducing the manual effort required for complex data analysis and ensuring that users can focus on their research rather than on data preparation intricacies. Moreover, the tool actively reports errors and guides users in correcting them, ensuring that only valid inputs proceed to analysis. Importantly, throughout the algorithmic pipeline, users are presented with additional options, such as the ability to exclude specific samples from the normalization process by pinpointing them via their unique identifiers. This feature is particularly vital for preserving samples deemed crucial for research, thereby avoiding their potential exclusion by the algorithm. This attention to detail in managing data integrity before and after equalization processes is a testament to the software’s reliability and the development team’s commitment to delivering a quality tool.

### 3.2. Concise and Easy Reporting Features

ArsHive’s reporting feature is a cornerstone of its user experience, seamlessly blending in-depth analysis with user accessibility. At the outset, the report is designed to be easily interpretable, incorporating examples to aid in understanding the results, a thoughtful addition that eases the learning curve often associated with similar software tools. This approach is particularly beneficial in the context of biomedical research, where clear and concise data interpretation is crucial.

The report’s structure facilitates a comprehensive understanding of the data. It presents before-and-after comparisons, allowing users to assess the impact of the varied algorithms on their dataset. Each variable is carefully analyzed, with the correct statistical tests being applied, resulting in corresponding *p*-values, among other relevant results, in order to offer an insightful glimpse into the dataset’s nuances, making it invaluable for in-depth analysis.

It also addresses the often-overlooked aspect of missing values. The report not only identifies these values but also quantifies them, ensuring that users have a complete overview of their data’s integrity. Furthermore, the software ranks variables based on their influence on the normalization process and provides metrics, such as total processing time (ArsHive was constructed as is suited for computers with lower processing power) and the counts of removed and surviving samples. This information is crucial for understanding the algorithm’s efficiency and the dataset’s dynamics post-processing.

Another critical feature of the report is the data integrity assurance. ArsHive performs a sanity check to ensure there is no overlap between deleted and surviving datasets, reinforcing the reliability of the results. Finally, the report concludes with comprehensive tables that detail counts, proportions, and percentage changes. These tables serve as a definitive reference for understanding an algorithm’s effect on a given dataset.

**Figure 4:**
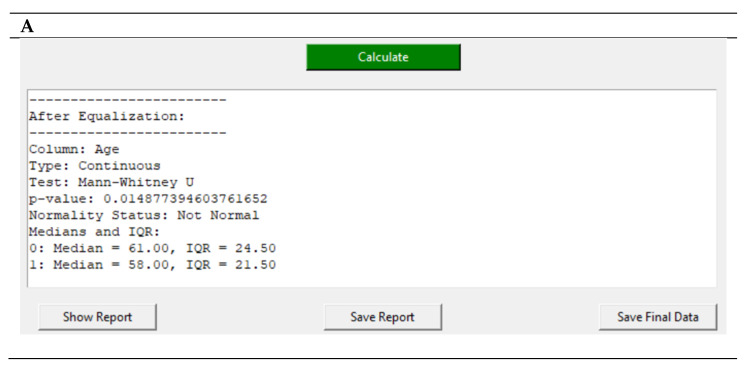

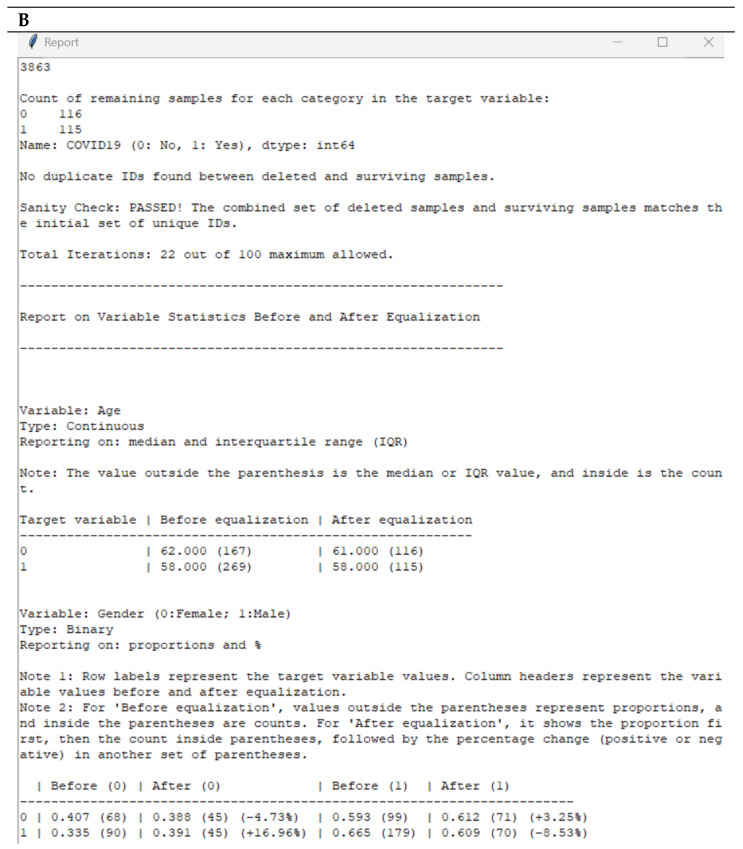
Comprehensive reporting in ArsHive. (**A**) Integrated report view within the GUI; (**B**) a snippet of the dedicated report window, providing detailed insights and comparison of data pre- and post-normalization.

For enhanced user convenience and collaboration, the reporting feature includes the ability to save the complete report as a formatted .txt file. This functionality is crucial for facilitating the sharing and discussion of results in research teams. To provide readers with a clear view of the reporting capabilities within ArsHive, please refer to Figure 4A, showcasing the main GUI’s reporting interface, and Figure 4B, depicting the dedicated reporting window. These illustrate the depth of analysis and the comprehensive nature of the reports generated by ArsHive. The complete report provides statistical analyses, test results, variable impacts, and data integrity details. It also provides before and after comparisons of data normalization, shedding light on the statistical significance and effect size of the analysis. Notations within the report guide the reader through the data, ensuring clarity in interpretation and facilitating scholarly dialogue.

In the present work, three distinct datasets were analyzed, with each uniquely constructed to demonstrate the versatility and efficiency of ArsHive. Biomedical dataset 1 is smaller, encompassing basic demographic and clinical variables alongside the target variable. The subsequent biomedical datasets, dataset 2 and dataset 3, scale up in complexity; the second dataset includes a broader range of variables and samples, while the third incorporates FTIR spectroscopic data, in the mid-infrared region. Notably, while the processing times across these datasets showcase our code’s optimization, the variation in token usage is inherently influenced by the dataset’s sizes. This is an essential factor, especially when considering the use of A.D.A., our digital assistant, in Section 3.3. Table 3 provides a concise summary of these datasets, illustrating their impact on processing efficiency and resource utilization, with a particular focus on the variance in token numbers dictated by dataset size. All datasets made use of the most advanced equalization algorithm for direct comparison purposes.

Let us consider dataset 3 as an example, with the presence of Coronavirus Disease 2019 (COVID-19), caused by the Severe Acute Respiratory Syndrome Coronavirus 2 (SARS-CoV-2), as the ‘target variable’, and considering demographic variables, such as age and gender, along with clinical or therapeutic variables, including death in the ICU, invasive mechanical ventilation (IMV), and extracorporeal membrane oxygenation (ECMO). The reporting generated by our software quickly and easily allows us to create, e.g., a table that summarizes these variables, as shown in Table 4. Additionally, it provides the count numbers for each variable, their respective proportions, and includes the statistical test used along with its corresponding *p*-value. Furthermore, the software offers detailed information on the samples considered or discarded in each run, type (continuous or categorical variable), their normality status (relevant for continuous variables), missing values per variable, and the total time taken for processing, among other insights.

Regarding the estimated number of tokens, their relevance, as mentioned previously, will become more apparent in Section 3.3. However, for now, it is important to understand the methodology used for calculating these token estimates. There are, admittedly, several approaches to token counting in datasets. One could use OpenAI’s ‘GPT-2 tokenizer’ from an earlier GPT model iteration [37]. However, this method faces challenges with complex datasets, like ours, which comprise a mix of strings (such as letters and words), integers, and floats (numbers or decimals), rather than just textual data. To simplify this process, we adopted an approach where each cell in the dataset is assumed to contain the word “text”. This allows us to simply multiply the number of tokens (in this case, one word per cell) by the total number of rows and columns in each dataset. We rely on OpenAI’s guideline for language models, which equates 1000 tokens to approximately 750 words. This ratio gives us a value of 1.333 tokens per word. Based on this assumption, we calculated the estimated number of tokens for each dataset, as shown in Table 3. 

In summary, ArsHive’s reporting feature exemplifies the software’s commitment to delivering a user-friendly yet comprehensive tool for biomedical informatics research. By providing detailed, accessible reports, it aids researchers in the critical task of data interpretation and sharing, furthering the pursuit of scientific discovery.

### 3.3. A.D.A.—A Digital Assistant Companion Tool 

**Figure 5:**
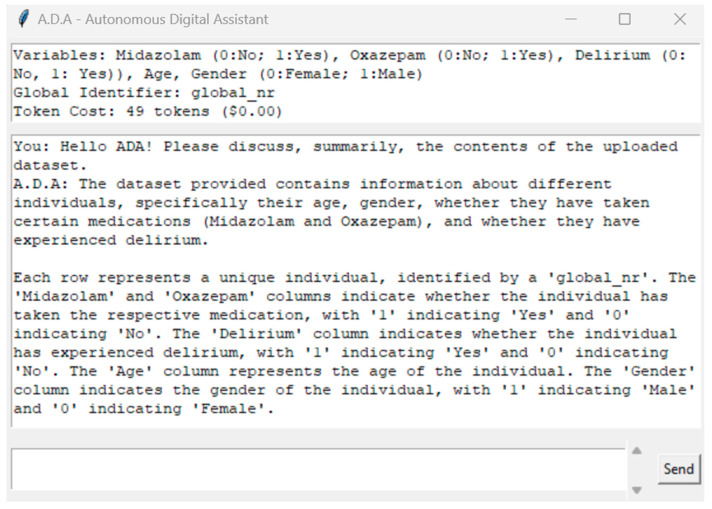
Interface overview and cost estimation with A.D.A. in ArsHive.

The development and integration of A.D.A. within our ArsHive software suite represent a significant step forward in harnessing AI to enhance biomedical research workflows. A.D.A. is not merely a feature; it is a sophisticated companion tool designed to assist researchers by parsing complex datasets and providing insightful guidance throughout the data analysis process. While we aspire to eventually incorporate our own AI-powered solutions or leverage publicly available algorithms that employ deep learning and large language model capabilities, we recognize the value and, albeit limited, impact of using OpenAI’s models at present. Therefore, we have equipped ArsHive with the option for users to utilize tools, like GPT-4, should they choose to do so, thus affording flexibility and choice in their research methodologies. Upon activating A.D.A., researchers are presented with an interface that clearly delineates their loaded dataset. Figure 5 captures this moment, illustrating A.D.A.’s GUI, in which the dataset’s variables for dataset 1 are identified. For usage costs (calculated by tokens used), we refer the reader to OpenAI’s official API documentation [38]. These insights are essential for efficient interaction with the digital assistant, especially when being mindful of the token processing limits outlined by the GPT-4 API constraints.

As highlighted in Section 3.2, token economy is a critical consideration. Our biomedical dataset 3, containing over 2 million tokens—primarily from the extensive FTIR data—necessitates the user-guided elimination of non-relevant variables to focus on the most pertinent clinical or therapeutic data. This curation step is crucial before engagement with A.D.A., ensuring that only relevant information is analyzed, thus maximizing interaction efficiency.

We should clarify that the token limits imposed by OpenAI’s API are separate from the GPT-4 model’s capabilities. OpenAI’s API server rate limits serve as a cap on the number of tokens that can be processed per minute, as well as the model’s maximum context length, which for gpt-4, for example, is 8192 tokens; our version within A.D.A., gpt-4 turbo, boasts a much-improved limit of 128,000 tokens. Regardless of the model used, it is important to note that this constraint may impact the assistant’s ability to handle large datasets, such as biomedical dataset 3, in a single interaction. 

Despite these limitations, A.D.A. remains a powerful ally for researchers. The digital assistant offers a wealth of knowledge and capabilities, from proposing solutions and suggesting code snippets for statistical problems to acting as a first reviewer of sorts. This assistance is invaluable, complementing the user’s expertise and enhancing the research process.

Looking ahead, the challenges and opportunities for A.D.A., and the ArsHive software at large, are significant. The subsequent section, Challenges and Considerations, will explore these topics more in depth, outlining our roadmap for development and the goals set out for our software suite.

## 4. Challenges and Considerations

Like any newly developed tool, ours had its challenges, which also presented opportunities for further development. One of the core challenges lies in maintaining the delicate equilibrium between granting users the freedom to explore data and safeguarding them from the complexities that may overshadow the utility of the software. This balancing act is central to preserving the essence of ArsHive—providing expertise in areas where users may lack depth.

The implementation of the (user-optional tool) A.D.A. digital assistant, leveraging the capabilities of GPT-4 and OpenAI’s API, underscores another concern: data protection and privacy. Despite datasets being locally stored, the requirement to transmit data to OpenAI’s servers for processing through LLMs raises questions about the future of data security. We are exploring avenues to empower users with the option to deploy their own LLMs on private servers or more robust systems, ensuring greater control over their sensitive data. A major external constraint with the use of A.D.A. comes from the current limitations of OpenAI’s API, particularly in the context input and output token length, as well as the tokens per minute (TPM) limit. These restrictions pose a significant challenge, as typical large biomedical datasets exceed these limits, necessitating a compromise from users to focus on the most pertinent data for analysis. 

The integration of OpenAI’s API into ArsHive is a strategic move to provide users with advanced LLM capabilities without the need for expensive subscriptions. Utilizing the API allows for a *pay-as-you-go* model, ideal for smaller research groups that require flexibility in managing their resources. As for the future of our tool, it is envisioned with several enhancements. As we tread this path, we anticipate the democratization of technology to be the ultimate culmination of our efforts. 

Table 5 presents a dual perspective of our journey: it lists key challenges encountered in the development of ArsHive and A.D.A. on the left, alongside a range of considerations and initiatives for future work on the right. These entries are not paired as direct solutions to their corresponding challenges but rather as independent elements that contribute to our overall vision for the evolution of ArsHive.

**Figure 6:**
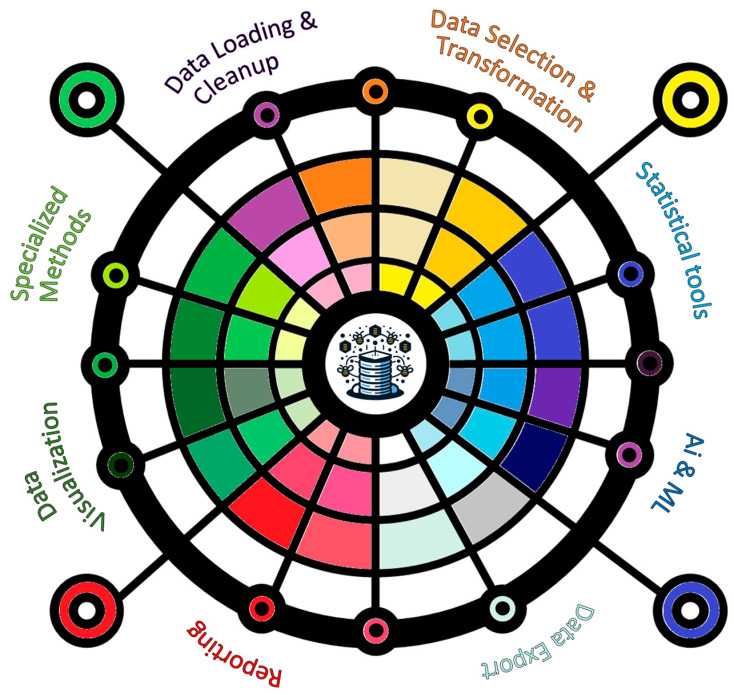
Conceptualization of future GUI.

This trajectory is further conceptualized in Figure 6, depicting a potential redesign of the GUI interface, moving towards an ecosystem that facilitates a broad spectrum of research methodologies and data types. In it, the user can choose what is displayed at any given time, and how the various tools and methods interact with each other, not unlike puzzle pieces. For example, the user could select the demographic variables in the corresponding *widget* and directly connect it to a visualization and reporting tool, in a single drag and drop mode. One of the objectives with this revamped GUI would be to allow for near infinite possibilities to pre- and process data, and to allow a much faster path from loading the data to reporting it directly into a manuscript to be evaluated by peers.

## 5. Final Remarks

In an era where the complexity of biomedical research is escalating, there is a growing necessity to streamline workflows and expedite the preprocessing and processing of datasets. Researchers are often required to navigate areas beyond their core expertise, underscoring the need for tools that simplify and automate these processes. ArsHive, in its developmental stage, is designed with the intent to be a user-friendly, educational, and automated solution for handling complex biomedical data. It aims to demonstrate that powerful analytical tools can be intuitive and accessible, broadening the scope of research capabilities across various disciplines.

At its current stage, ArsHive is being tested to validate its ability to offer seamless data processing and analysis, insightful reporting, and integration with the A.D.A. companion tool, powered by the latest advancements in artificial intelligence. These features are intended to empower researchers to tackle data challenges with greater efficiency and understanding. However, ArsHive, developed by a small yet dedicated research team, faces challenges, such as limited resources and reach, and external constraints, like the API limitations of third-party services. These hurdles motivate our continued commitment to improvement and innovation. 

Looking forward, we envision ArsHive evolving into a more sophisticated tool that integrates seamlessly with established platforms, thereby enhancing its applicability across scientific domains. To support this objective, our roadmap outlines strategic enhancements to the user interface, robust privacy safeguards, and the expansion of educational capabilities, ensuring that ArsHive remains at the forefront of democratizing advanced technological tools for researchers.

As development progresses, our goal remains to equip the scientific community with a tool that facilitates research and actively contributes to discovery and innovation. Thus, ArsHive is positioned as a dynamic companion in the ongoing journey of biomedical research, with its full potential yet to be realized and validated through continued user feedback and enhancements.

## Figures and Tables

**Table 1:** Overview of publicly available human-specific biomedical datasets.

Dataset	Type	Brief Description	Size	Ref.
MIMIC-IV	Clinical	Patient data including physiological variables, treatments, and diagnostics, in the intensive care unit (ICU).	>60,000 ICU admissions	[31]
eICU Collaborative Research Database	Clinical	Multi-center including vital signs, laboratory work, and Acute Physiology and Chronic Health Evaluation (APACHE) score.	>200,000 ICU admissions	[32]
HiRID	Clinical	High-resolution ICU dataset with demographic data and detailed treatment parameters.	>30,000 ICU admissions	[33]
Georgetown Immuno-oncology registry	Clinical and genomic	Electronic health records including demographic, and clinical, prescription information and retrospective outcomes research at the 10 DC-Baltimore based MedStar Health network hospitals and Hackensack Meridian Health system in New Jersey.	N/A	[34]
NCBI	Genomic	A collection of databases for biotechnology and biomedicine, including nucleotide sequences, protein sequences, and literature. Size varies by database (e.g., GenBank, PubMed).	Extensive	[35]
Quartet metabolomics Project	Metabolomics	Metabolite reference materials from B lymphoblastoid cell lines for inter-laboratory proficiency testing and data integration of metabolomics profiling.	N/A	[36]

**Table 2:** Automated statistical tests available within ArsHive *.

Variable Type		Comparisons between 2 Groups	Comparisons between 3 or More Groups
Continuous variables	Parametric	Two-sample *t*-test	ANOVA
	Non-parametric	Mann–Whitney U test	Kruskal–Wallis one-way ANOVA test
Categorical/ nominal variables		Chi-square test or Fisher’s exact test (if the applicability conditions of the first test are not verified)	Chi-square test or Fisher’s exact test (if the applicability conditions of the first test are not verified)

* Post hoc not represented.

**Table 3:** Comparison of the different characteristics for the different biomedical datasets.

Characteristic	Dataset 1	Dataset 2	Dataset 3
Number of demographic variables	2	3	3
Number of clinical/therapeutical variables	2	18	10
Total number of rows (samples)	26	225	436
Total number of columns (variables ^#^)	6	29	3750
Total processing time (in seconds *)	2.06	2.78	34.06
Estimated number of tokens	173	8398	2,178,874

^#^ including the target variable,* for the most advanced normalization algorithm.

**Table 4:** Dimension of the populations used (COVID-19 and control group), with corresponding *p*-value of the statistical tests comparing the two groups.

Variable		COVID-19 (*n* = 112 Patients)	CONTROL (*n* = 103 Patients)	*p*-Value
Gender (*n*/proportion)	Female	44 (0.39)	40 (0.39)	1.000 *
	Male	68 (0.61)	63 (0.61)	

Age (years), median (IQR)		59 (21)	62 (23)	0.117 ^●^

UCI death (*n*/proportion)	No	89 (0.79)	79 (0.77)	0.745 *
	Yes	23 (0.21)	24 (0.23)	

IMV (*n*/proportion)	No	36 (0.32)	33 (0.32)	1.000 *
	Yes	76 (0.68)	70 (0.68)	

ECMO (*n*/proportion)	No	104 (0.93)	95 (0.92)	1.000 *
	Yes	8 (0.07)	8 (0.08)	

Statistical tests used: ^●^ MWU (Mann–Whitney U) and * χ^2^ (Chi-square).

**Table 5:** Summary of key challenges and future considerations for ArsHive and A.D.A.

Challenges	Considerations/Future Work
Balancing user autonomy with guidance	Educational empowerment with toggles and feedback options
OpenAI’s API token and TPM limitations	GUI enhancements for visual data manipulation
Data privacy concerns with cloud processing	Integration with established platforms or standalone option
Need for local LLM deployment options	Quality assurance and advanced GUI overhaul
Economical access to LLM capabilities	Privacy-by-design approach with no data retention
Ensuring relevance in data curation for ADA	Community collaboration and continuous software development

## Data Availability

The original contributions presented in the study are included in the article. Further inquiries can be directed to the corresponding author/s.
